# Growth of Floc Structure and Subsequence Compaction into Smaller Granules through Breakup and Rearrangement of Aluminum Flocs in a Constant Laminar Shear Flow

**DOI:** 10.3390/gels10010049

**Published:** 2024-01-10

**Authors:** Mii Fukuda Hayami, Takashi Menju, Takeshi Ide, Tatsuro Uchida, Yasuhisa Adachi

**Affiliations:** 1 Infrastructure Systems Research and Development Center, Toshiba Infrastructure Systems & Solutions Corporation, 1, Toshiba, Fuchu 183-8511, Tokyo, Japan; 2 Energy Systems Research and Development Center, Toshiba Energy Systems & Solutions Corporation, 1-20, Kansei, Tsurumi, Yokohama 230-0034, Kanagawa, Japan; 3 Faculty of Life and Environmental Science, University of Tsukuba, 1-1-1, Tennodai, Tsukuba 305-8572, Ibaraki, Japan; adachi.yasuhisa.gu@u.tsukuba.ac.jp

**Keywords:** PACl, aluminum hydroxide gel, ALT ratio, flocculation, densification, erosion breakup

## Abstract

We have constructed an outer-cylinder-rotating Couette device for high-speed shear flow in laminar flow conditions and visualized the structure formation and subsequent rearrangement of PACl (flocculant made of aluminum hydroxide gel) and kaolinite flocs by visible light imaging. In a previous report, we analyzed the case of relatively low shear rate (*G*-value = 29 1/s) and confirmed that the flocculation process could be separated into two stages: a floc growth stage and a breakup/rearrangement stage. Once the large bulky flocs that reached the maximum size appeared, they rearranged and densified through structural fracture and rearrangement. In this report, this process was further investigated by conducting experiments under two different high shear rates (58 and 78 1/s) at which breakup and rearrangement became more pronounced, and three different aluminum kaolinite ratios (ALT ratios) that were over and under the optimum dosage (neutralization point by Zeta potential). Visualization results confirmed that, during the growth stage, the flocculation rate could be approximated by a scaling relationship between floc size and elapsed time, which depended on the ALT ratio. After reaching the maximum size, the floc rapidly became compact and dense following adsorption of the gel, incorporating fine fragments from erosion breakup. The over and under dosages created a lot of fragments of erosion breakup, but less so in the optimum dosage. In the optimum ALT ratio, fragments did not remain because they were incorporated into the flocs and densified, and the floc size was thought to be maintained. The floc circularity distribution peaked at around 0.6 and 1, suggesting that the flocs were spherical in shape due to erosion breakup.

## 1. Introduction

Flocculation is an important initial stage in colloidal gel formation. The incorporation of many functional chemicals during the initial stages of gelation and the eventual formation of compact structures are critical to establishing new material processes. In the case of water treatment, it can improve separation efficiency and reduce the amount of bulky sludge. When a flocculant is added to treated water containing suspended particles and the solution is agitated, the initially dispersed fine suspended particles collide and adhere to each other, inducing the development of large flocs that can be easily identified by the naked eye. At this point, it is necessary to develop the technology to form flocs that are easily separated according to the mechanism of solid–liquid separation (sedimentation, dewatering, membrane filtration) which will be carried out in a later stage of the operation. For this purpose, clarification of the physical properties of flocs, such as size and strength, in relation to flocculation conditions—going back to the floc formation process—is warranted, and many studies exist on the flocculation rate, floc formation process, sedimentation rate, and flow characteristics of turbid water containing flocs [1]. The kinetics of the initial stage of flocculation has been analyzed theoretically as a subject of fundamental physics long ago [2]; however, from an engineering standpoint, issues have been considerably addressed by the field of water treatment.

One of the basic experiments was reported by Tambo et al. [3] using kaolinite suspension to flocculate with aluminum sulfate. The flocs were grown by rapid agitation and mixing followed by slow agitation, which mimics actual water treatment. The formed flocs were taken out in a single state and the settling behavior of the flocs was photographed as a function of floc diameter. By applying Stokes’ law, the authors recognized that the density of the floc effective sedimentation decreased with increasing floc diameter following a power-law relationship. The relation was named the floc density function. This means that there is a certain law of floc density as a function of floc diameter in the seemingly random structural formation of flocs. Furthermore, Tambo continued his experiments and organized the floc density function in terms of the ALT ratio, which is the ratio of the concentration of aluminum ions in the flocculant to the concentration of suspended particles, and pointed out that the floc morphology changes systematically according to chemical conditions [4]. On the other hand, Kusuda [5] reported that differences in agitation intensity during floc formation affected the floc density function, pointing out the importance of agitation intensity in the formation of floc structure. However, the interrelation between the results of Tambo and Kusuda has not been investigated.

In the 1980s, many papers on the structural formation of flocs were published as a subject of fractal analysis based on the concept according to which flocs were formed by the binary collision of clusters [6]. By means of numerical methods, Meakin [7,8] demonstrated occurrence of cluster rearrangement, the importance of which has also been demonstrated experimentally [9,10,11,12,13]. The prediction of floc size is important for the rational design of the separation process and has received substantial attention, alongside floc strength, since the early days of the research process [14] and many articles have been published which address, experimentally and numerically, the breakup of flocs in a simple system [15,16,17,18,19,20,21,22,23,24] and real systems. There are two types of fracture mechanisms: large-scale fragmentation due to tensile force and surface erosion due to shear force [25].

However, the mechanisms of such destruction and the issue of incorporating inter-cluster rearrangements into the floc formation process remain unclear despite their importance [26,27,28]. Serra et al. [29] monitored salt aggregation of latex particles for a long time with a vertically installed Couette device with a rotating inner cylinder. Their results revealed evidence of cluster rearrangement. On this problem, Selomulya et al. [30,31,32] have reported extensively to demonstrate breakup and rearrangement of clusters using latex suspension and have proposed the analytical method of population balance. Their results clearly show that the size of the floc increased in the beginning, and in some cases, the size decreased after reaching the maximum. However, the condition and reproducibility of their behavior was not fully analyzed. The effectiveness of this method was reviewed by Jeldres et al. [33] by placing emphasis on the permeability and restructuring process. A variety of studies entailing not only simple shear but also programmed, well-defined flows have been published recently, revealing the frequent appearance of densification processes during flocculation [34,35]. However, such results are not fully reflected in the design of water treatment processes; therefore, the original question of the effect of the ALT ratio on flocculation remains unanswered.

Conventionally, in water purification plants, the main control factors for the flocculation process are the injection rate of the flocculant (PACl) and pH. To increase floc size, flocculation is generally performed with rapid and slow fixed intensities. Large flocs have a higher settling velocity related to the separation load in the settling basin. However, the relation between floc size and settling velocity is complex, and, as the floc grows according to the fractal scaling law, the density decreases with respect to the floc size, following a power-law relationship. Therefore, the authors attempted to develop a progressive flocculation method able to achieve high density and large flocs to simplify solid–liquid separation facilities. We investigated the possibility of high-density and high-settling-velocity flocs by controlling not only PACl and pH, but also agitation intensity. In a previous report [36], the ratio of kaolinite to PACl was fixed under a laminar shear flow (*G*-value 29 1/s) to visualize the structural changes in the floc. As a result, the flocs grew rapidly in the initial stage of flocculation but did not grow beyond a certain diameter and were broken up by the flow field. Surrounding fragments produced by the fracturing were incorporated into larger flocs and rearrangements, contributing to densification. Thus, under constant agitation intensity, the flocs were qualitatively confirmed to increase in density through repeated growth, fracture, and rearrangement. It was confirmed that these phenomena tend to occur under a higher shear flow.

The purpose of the present investigation is to obtain the characteristics of the floc structure under a constant laminar shear flow by varying the ALT ratio and agitation intensity simultaneously. A further objective is that of understanding the details of the phenomenon of floc densification by rearrangement and determining the conditions under which flocs are easily densified.

## 2. Results and Discussion

The horizontal Couette device with a rotational outer cylinder was used to fill the gap between the inner and outer cylinders with kaolinite and PACl test water to generate shear force in response to the number of rotations. The agitation strength in the gap was defined as *G*, and the representative floc diameter distribution was defined as *D*_90_. A total of six conditions were tested: two rotational speeds of 120 rpm and 160 rpm, and three ALT ratios, the ratio of aluminum to kaolinite in PACl (shortage—0.03 and excess—0.10 relative to the optimal dosage—0.05, which is charge neutralization by zeta potential). Temporal changes in constant laminar shear flow and floc structure were measured by image photography. Details of the test equipment, image and data analysis are described in a previous report [36] and Section 4.

### 2.1. Floc Images and Structual Change

**Figure 1:**
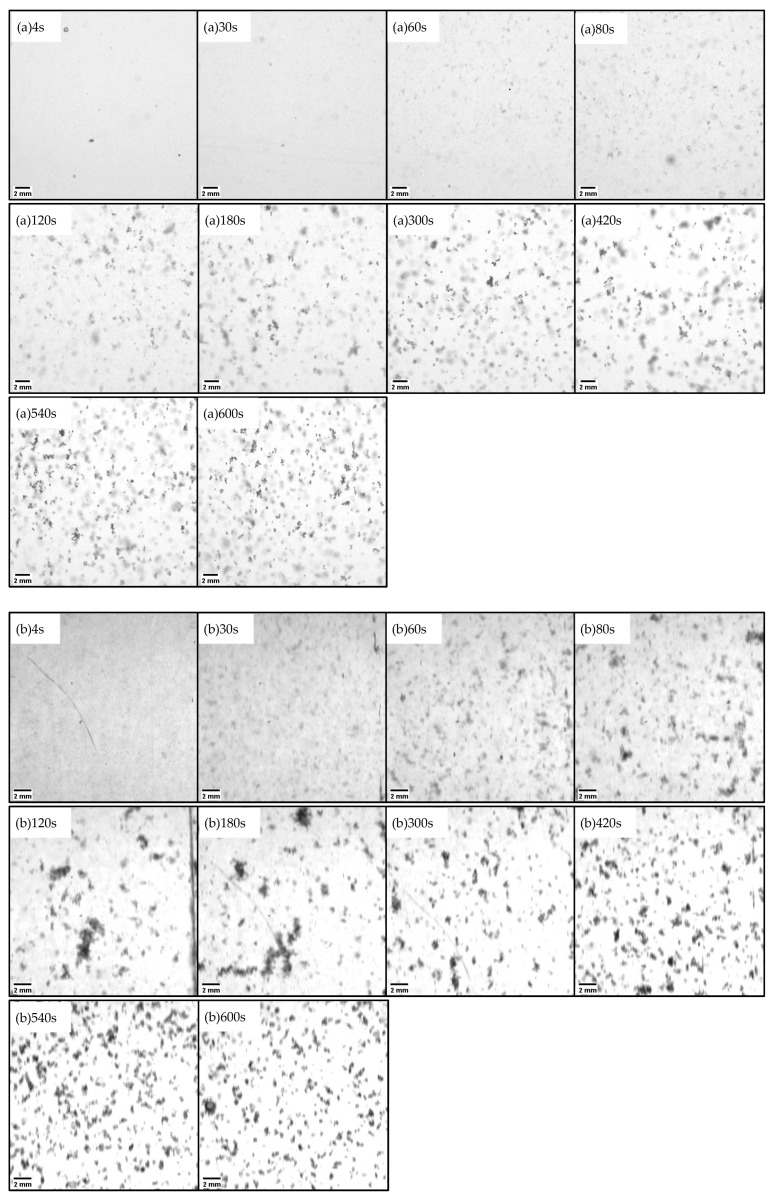

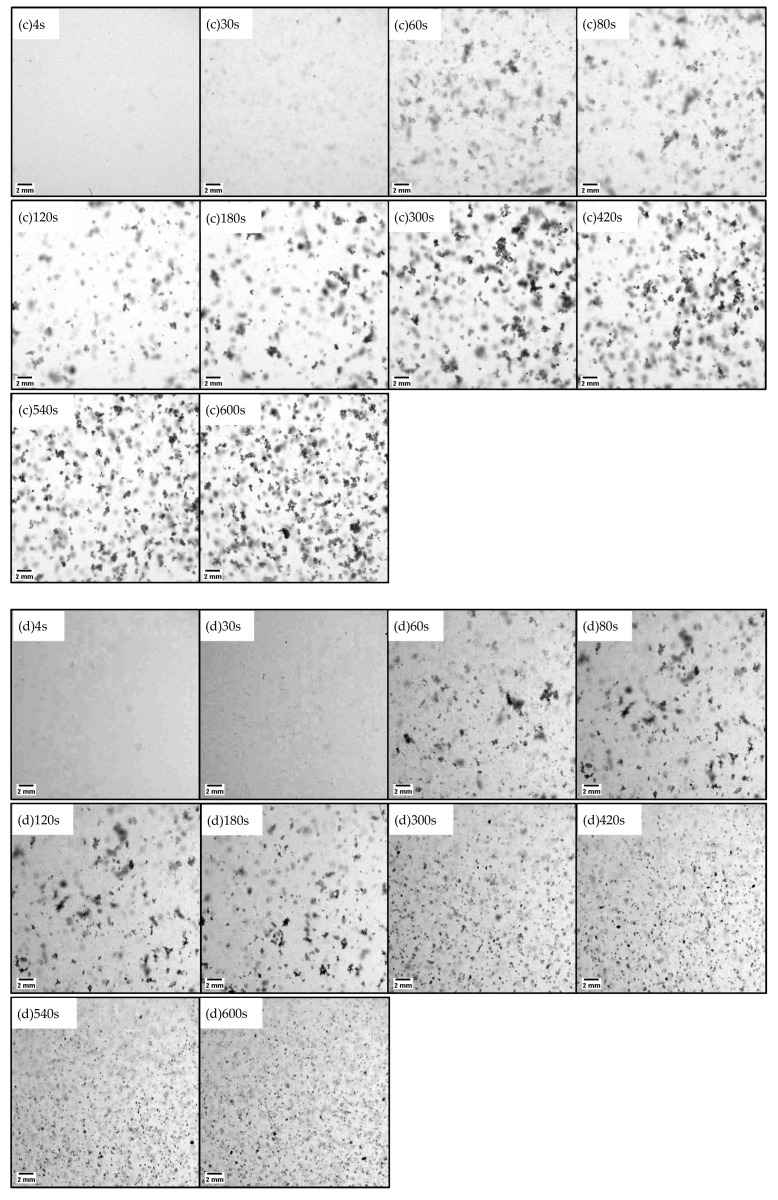

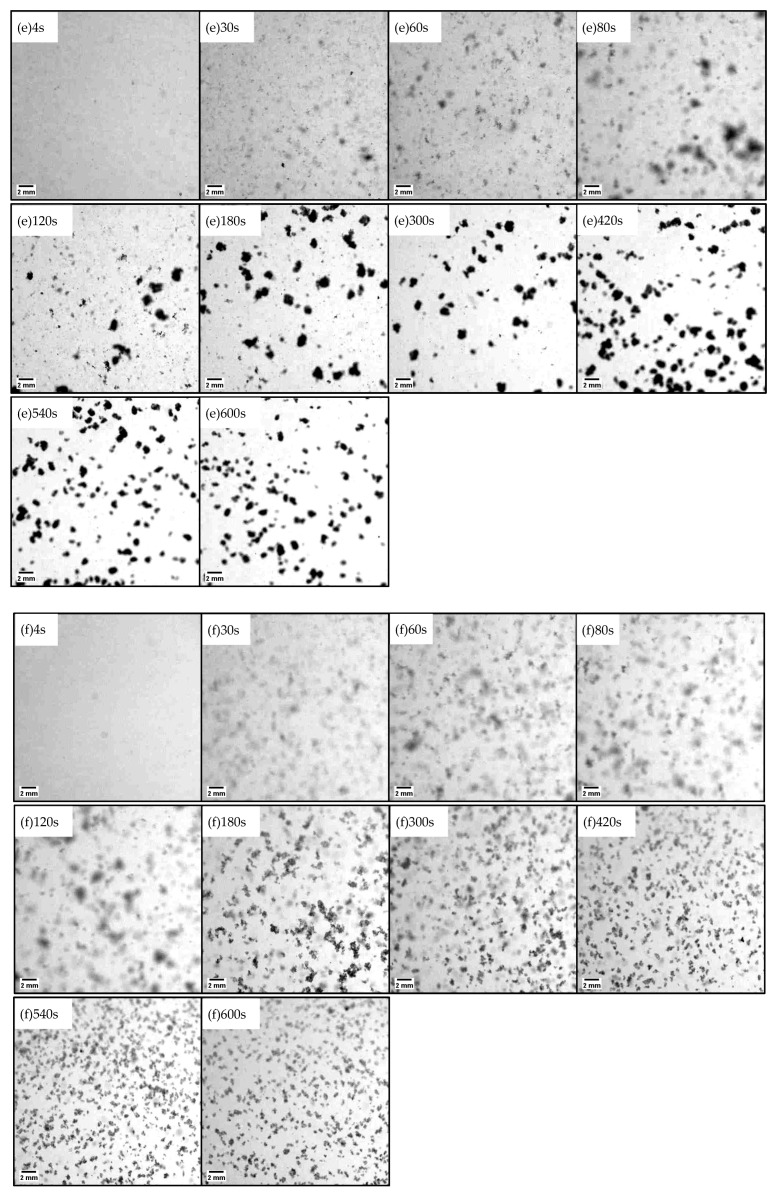
Photographed images of flocs in six tests in which the flocculation conditions are changed to two different rotation speeds and three different ALT ratios: (**a**) 120 rpm, ALT ratio 0.03, (**b**) 120 rpm, ALT ratio 0.05, (**c**) 120 rpm, ALT ratio 0.10, (**d**) 160 rpm ALT ratio 0.03, (**e**) 160 rpm, ALT ratio 0.05, and (**f**) 160 rpm, ALT ratio 0.10. The horizontal bar in the bottom left corner of each image is a 2 mm scale bar. Figure 1 includes RGB original images converted to 16-bit for confirmation by the naked eye. For image analysis, the original RGB images were converted to 8-bit according to the specifications of the analysis software.

Figure 1a–f shows images taken for test conditions at varying ALT ratios, from 0.03 to 0.05 and 0.10, and at rotational speeds of 120 rpm and 160 rpm. For each test condition, images were taken 4, 30, 60, 80, 120, 180, 300, 420, 540, and 600 s after the test had commenced. Figure 1 includes original RGB images converted to 16-bit to easily confirm the progress of floc aggregation with the naked eye. For image analysis, the original RGB image was converted to 8-bit. In Figure 1, the original images of 1024 pixel squares were cropped to 512 pixel squares including the ROI (region of interest) for easy comparison of each condition. This was a characteristic of background illumination: kaolinite did not transmit light and therefore appeared black, while the gel portion, which was insoluble aluminum hydroxide, was transparent and did not appear in the image. Therefore, as shown in a previous report [36], when the floc density was small, the kaolinite in the floc was dispersed and appeared to be light gray in color, while when the floc density was high the kaolinite was concentrated and appeared to be dark black. The brightness of the flocs afforded a qualitative understanding of the density transition of flocs. As the rotation continued, indistinct flocs with light gray contours began to form. Gradually, the flocs grew, and multiple large bulky flocs were observed to be present. After the flocs reached their maximum diameter, further continued rotation caused the bulky flocs to disappear. There were two types of flocs: those that destroyed and refined, and those that incorporated the surrounding micro flocs and underwent a dense structural change (rearrangement). At the time of the disappearance of the former bulky flocs, the flocs had become smaller in size due to separate breakup of the flocs. In the latter case, shear forces caused erosive breaking of the floc surface, and fine fragments were incorporated into other flocs and densified.

**Figure 2:**
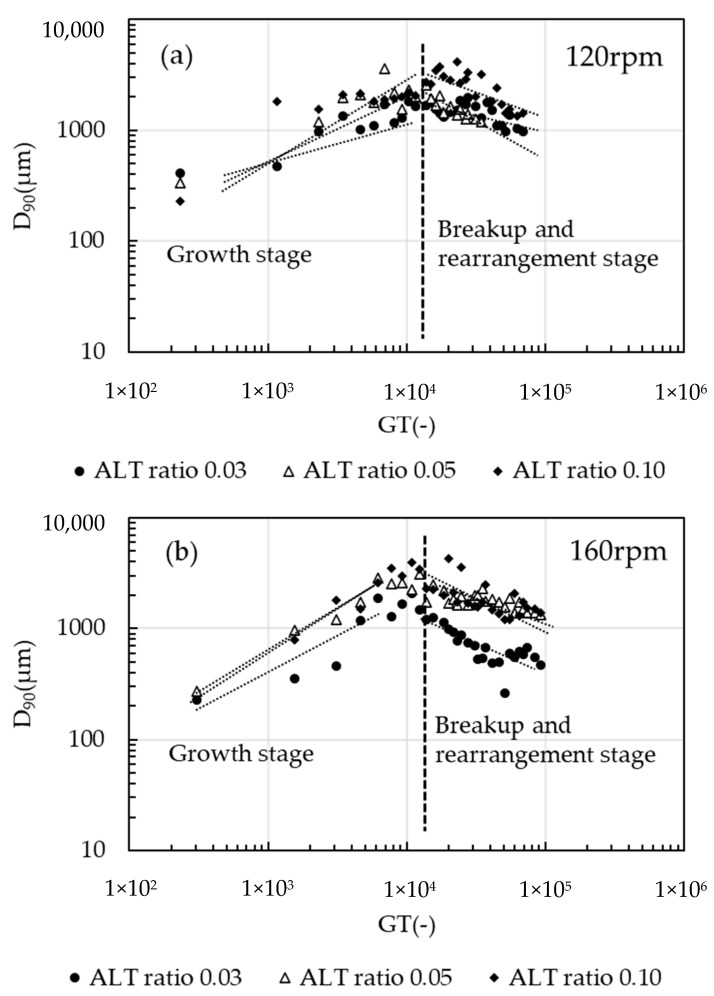
Log–log plots of *D*_90_ vs. *GT* values, (**a**) 120 rpm and (**b**) 160 rpm conditions. *D*_90_ increases as a power of the *GT* value in the growth stage and tends to decrease after a certain *GT* value. *D*_90_ decreases by a power in the breakup and rearrangement stage.

Figure 2a,b shows the logarithmic relationship between *D*_90_ and *GT* values, i.e., the result of image analysis. The *GT* value on the horizontal axis is the product of the agitation strength (shear rate) *G* value and the elapsed time. *D*_90_ increased following a power-law relationship with *GT*, and then decreased after a certain point around 13,000 *GT* value. This trend was also observed in other test conditions. The floc growth stage and the breakup and rearrangement stage were clearly separated by a certain *GT* value.

### 2.2. Floc Growth and Breakup/Rearrengement Stages

All data from Figure 1 and Figure 2 were classified into floc structure growth stage and breakup/rearrangement stage, regardless of the *G* value (shear rate) and ALT ratio. The boundary between growth and breakup was around *GT* = 13,000. Breakup was also evident, as small particles occurred after the appearance of the largest diameter. As the shear force continued to be applied, the floc diameter began to decrease, and the maximum diameter remained inconsistent. Near the maximum diameter, the floc became an irregularly shaped bulky floc, which then became compact. The flocs were spherical and exhibited compacting, and a peak existed at circularity 1. The double peaks were more clearly visible in the 160 rpm condition. Therefore, it is reasonable to divide the discussion into two phases: the floc structure growth stage and the breakup and rearrangement stage.

#### 2.2.1. Evaluation of Growth Stage

In every case, the relationship between *D*_90_ and *GT* can be approximated by applying a scaling law. The general floc diameter *D_f_* is a function of the *G* value in the growth stage [9,15]:

(1) $$D_{f}\approx G^{-0.5}.$$

**Figure 3:**
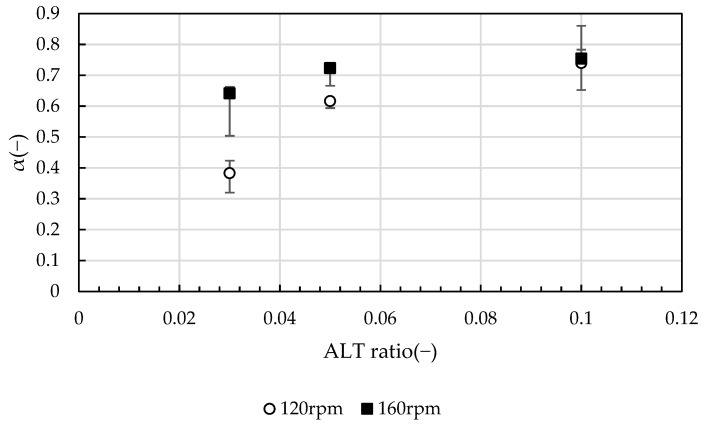
The power of *D*_90_ and *GT* values in the growth stage, *α*, represents the rate of flocculation. *α* increases with the ALT ratio. At the same ALT ratio, a lager *G* value is greater.

The stepwise decrease in *D_f_* with *G* value, as well as the change in the self-similarity structure is confirmed. Figure 3 shows the relationship between the slope *α* of *D*_90_ and *GT* values and the ALT ratio for the first 80 s of the test. The term *α* represents the flocculation rate and is positively related to the ALT ratio, with *D*_90_ increasing as a power of the ALT ratio. By comparing the same ALT ratio, the value of *α* was found to be greater at 160 rpm than at 120 rpm, and at an ALT ratio of 0.10 there was little difference in *G* values. From this trend, it is possible to speculate that the ALT ratio affects the flocculation rate, and for the same ALT ratio, conditions with a higher shear force (*G* value) applied to the floc tend to have a greater flocculation rate. Clearly, *α* is greatly influenced by the ALT ratio, followed by the *G* value. In the presence of an excess of flocculant, such as an ALT ratio of 0.10, the agitation conditions have little effect, and the ALT ratio dominantly determines the flocculation rate. At the optimum injection rate, the error bars are short and there is little variation in the data; at an ALT ratio of 0.10, the error bars are large and vary vertically. This reflects the larger size of the flocs and the variability of the flocs entering the analysis area. Tambo has shown a relationship in which the structural growth of flocs increases following a power-law relationship with respect to the ALT ratio in a two-stage mixing transition from rapid to slow mixing [3,4]. The present report also agreed that the aggregation rate varied as a power of the ALT ratio. Adachi et al. [37] also investigated the properties of flocs formed by increasing salt concentration in the flocculation of kaolinite and aluminum salts by measuring the sedimentation rate. In Adachi’s report, precipitated aluminum hydroxide contributed to the formation of large flocs, which were more pronounced near the charge neutralization of kaolinite. Such findings appear to agree with the present report in that *α* was found to increase as the flocculant concentration increased. This phenomenon can be considered as follows: the ALT ratio increases the concentration of aluminum hydroxide in the water. The flocculated flocs have a larger collision radius due to the attachment of aluminum hydroxide. Since the flocculation rate is proportional to the cube of the particle radius, *α* increases and the flocculation rate increases. At the same time, flocs with large fractal dimensions and low strength are formed, which become more fragile as the *G* value increases. In all conditions, growth occurred while maintaining fractality, but *α* showed different values for each condition. The reason why the *α* value of the growth stage varies with the *G* value is that breakup occurs at the same time as growth. The phenomenon of floc growth according to the fractal scaling law was more pronounced at an ALT ratio of 0.05 and 120 rpm, where *α* was close to 0.5. This shows that the growth rate and structure of the flocs can be controlled by adjusting the ALT ratio and *G* value.

#### 2.2.2. Evaluation of Breakup/Rearrangement Stage

In Figure 2, the breakup tendency was more clearly shown at 160 rpm than at 120 rpm. Also, when compared at the same *GT* value, the ALT ratio was less likely to breakup at the optimum value (near neutralization). Flocs that grow and nearly reach their maximum diameter are unable to keep their maximum diameter and become smaller by flow shear forces. It is expected that separative breakup will predominate during the growth stage, followed by erosive breakup during the breakup and rearrangement stage after the maximum diameter. Densification occurs as the small fragments generated by erosive breakup become incorporated into the surrounding larger flocs. In addition, erosion breakup is more frequent, and circularity is expected to increase. As circularity increases, the floc is expected to become denser under constant laminar shear flow due to periodic tension and compression associated with rotation. With appropriate control of the ALT ratio and *G* value, the flocs become granulated. This phenomenon was more pronounced at the 160 rpm condition, which is the optimum ALT ratio and had a higher shear force.

**Figure 4:**
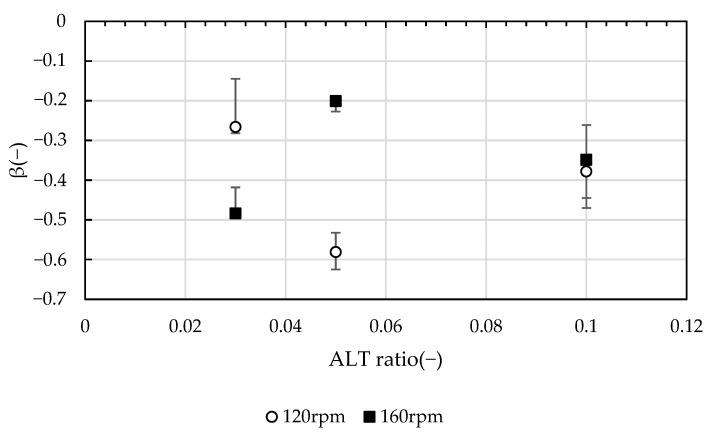
The power of *D*_90_ and *GT* value, *β*, represents the fracture rate; *β* is more influenced by the *G* value than by the ALT ratio.

Figure 4 shows the relationship between *D*_90_ and the *GT* values in the breakup and rearrangement stage after 180 s from the start of the test. The power of *D*_90_ and of the *GT* value, *β*, is the breakup/rearrangement rate at which the particle size decreases in the breakup/rearrangement stage. In this study, *D*_90_ decreased monotonically with respect to *GT* values, and the rate of decrease differed according to the ALT ratio and *G* values. The relationship between *β* and the ALT ratio was not as clear as in the growth stage, but, at ALT ratios of 0.03 and 0.05, *β* was more affected by *G* values than by the ALT ratio. Under the condition of an ALT ratio of 0.10, the difference in *β* by *G* value was small, and the ALT ratio was the rate-limiting factor for the aggregation reaction.

### 2.3. Floc Circularity

**Figure 5:**
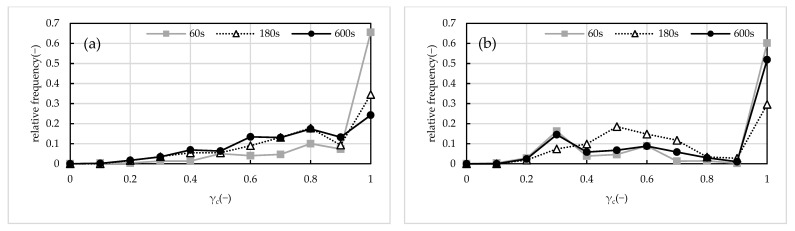

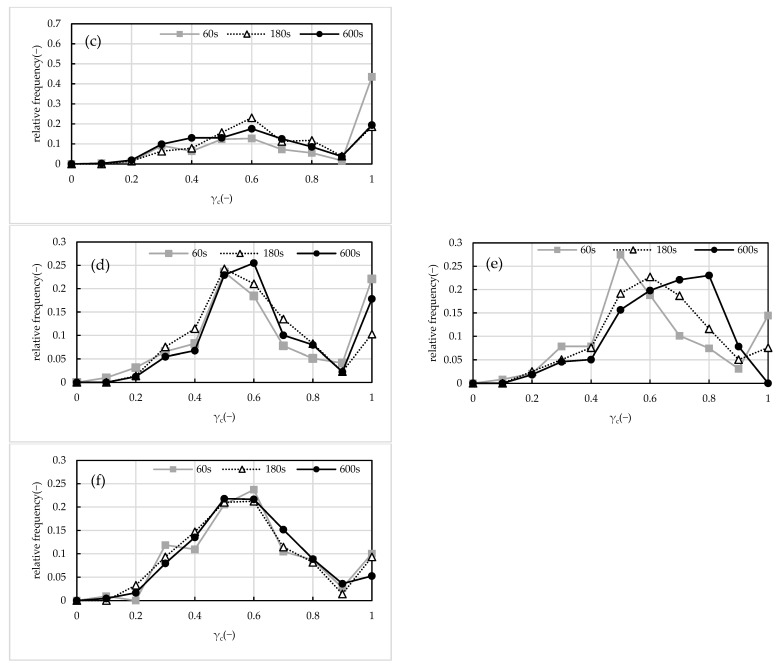
Relative frequency and temporal variation of circularity. (**a**) 120 rpm, ALT ratio 0.03, (**b**) 120 rpm, ALT ratio 0.05, (**c**) 120 rpm, ALT ratio 0.10, (**d**) 160 rpm, ALT ratio 0.03, (**e**) 160 rpm, ALT ratio 0.05, and (**f**) 160 rpm, ALT ratio 0.10. In (**b**,**e**), the approach to the circular shape was clear.

Figure 5a–f shows the change in circularity over time for each flocculation condition. The circularity per floc calculated by image analysis was statistically organized. The vertical axis indicates frequency normalized by the number of flocs, and the circularity distribution is presented at 60 s, 180 s, and 600 s after the start of test. As the floc grew and the bulky floc reached its maximum diameter, separate breakup occurred, and, as the floc became denser, erosion breakup occurred, with the floc becoming more circular. In conditions (b) and (e), where the ALT ratio was 0.05, it was quite clear that the circularity of the floc increased with elapsed time and approached a circular shape. Under the high shear conditions of (d–f), the circularity distribution had two peaks, circularity 1 and the rest. It always indicated the presence of spherical flocs. This means that the flocs with bulky structure reach their maximum diameter and the shape of the flocs approaches a spherical shape when erosion breakup is predominant.

### 2.4. Compactification of Floc Structure

**Figure 6:**
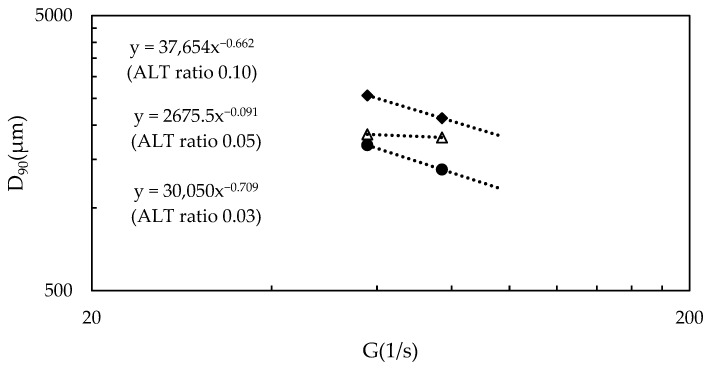
The relationship between *D*_90_ and the *G* value in the breakup/rearrangement stage is shown. To match the rpm conditions, image data were extracted at the timing when the *GT* value reached 13,000. For ALT ratios of 0.03 and 0.1, breakup was found to occur relative to the *G* value; for an ALT ratio of 0.05, breakup remains unclear, and the floc size was found to be maintained and densified.

Figure 6 shows the relationship between *D*_90_ and the *G* values for each of the ALT ratio for the breakup and rearrangement stage, *GT* = 13,000. At an ALT ratio of 0.03, the power was −0.70 and *D*_90_ decreased relative to the *G* value, indicating breakup; at an ALT ratio of 0.05, the power was very small (−0.091) and *D*_90_ was maintained. Lastly, at an ALT ratio of 0.10, the power was −0.66, indicating breakup as in the case of an ALT ratio of 0.03. This result indicates that, under conditions away from charge neutralization, the flocs are more likely to break with respect to the *G* value, whether the ALT ratio causes overdose or shortage. The unbalanced charge on the floc surfaces suggests that the flocs are slippery and more likely to fracture rather than bond. Under optimum ALT ratio conditions, *D*_90_ was found to maintain its size relative to the *G* value, with densification occurring without a pronounced breakup.

In the flow field, the flocs are densified and stabilized by incorporating small fragments under alternating compressive and tensile stresses. This phenomenon results in the formation of flocs with high intensity against high shear forces. As is evident from the results in Figure 5e, the circularity approaches 1 with time. The floc under this condition transforms structurally into a compact, dense, circular floc, with breakup and rearrangement occurring. In this report, we observed changes in floc structure occur under laminar flow conditions while maintaining the same *G* values.

As shown in this test results, the ALT ratio and *G* value affect the growth and breakup rate of flocs. The ALT ratio has a significant effect on the growth rate, and the structure of the flocs changed with the ALT ratio in the breakup/rearrangement stage. For the same ALT ratio, the higher the *G* value, the greater the growth rate. Therefore, the ALT ratio has a significant effect on the growth and breakup of the flocs. Furthermore, under the optimum ALT ratio conditions, floc breakup is not significant even with an increasing *G* value, and a compact structural change to high-density spherical particles was observed. The phenomenon of floc densification is clearly caused by the gelation behavior of the aluminum hydroxide gel. We postulate that the gel structure produced by insoluble aluminum hydroxide adsorbs and densifies the small fragments, and that erosion breakup forms even denser and more spherical flocs. This phenomenon was more pronounced under high-shear conditions (160 rpm). By comparing Figure 1d 600 s, Figure 1e 600 s, and Figure 1f 600 s, it is possible to observe that there are more fragments and smaller flocs in the shortage and overdosage ALT ratio conditions (Figure 1d 600 s, Figure 1f 600 s). Only Figure 1e 600 s, the optimum ALT ratio, has fewer fine fragments. Fragmentation due to the high density and compacting of the floc results from breakup. During the growth stage, fracturing occurs simultaneously with floc growth. Separate breakups predominate, resulting in relatively large clumps of flocs. These flocs, again, contribute to floc growth and form bulky flocs. In the fracture and rearrangement stage, erosion breakup is predominant, and small fragments are generated. The flocs incorporate these fragments and become denser and more compact.

High-density, spherical flocs have a high solid–liquid separation rate and improve water treatment efficiency. In this study, tests were conducted in a high-shear field to evaluate the structural changes in the floc, although confirmation is needed in a low-shear field as well. Additional data collection is important to investigate the relationship among the floc structure, the ALT ratio, and the *G* value.

## 3. Conclusions

Visualization tests of the kaolinite–PACl flocculation process (from initial flocculation to breakup and rearrangement) were conducted using the Couette device of the horizontal outer cylinder rotation type. In a constant laminar shear flow, the change of floc structures was observed by varying the ALT ratio and *G* value, resulting in the following findings:

- After the floc structure develops and becomes bulky, the floc becomes fine through breakup and rearrangement;
- The growth rate and breakup/rearrangement rate have a power-law relationship with respect to the *GT* value. Additionally, growth and breakup/rearrangement stages are clearly distinguishable around a certain *GT* value;
- Aggregation rate *α* during the growth stage increases with the ALT ratio. In an ALT ratio of 0.10, the effect of the *G* value is less significant;
- In the breakup/rearrangement stage, at ALT ratios of 0.30 and 0.50, *β* depends more on *G* values than on ALT ratios; at the ALT ratio of 0.10, *β* depends more on the ALT ratio than on *G* values;
- In the breakup/rearrangement stage, the floc diameter is maintained with respect to the *G* value under near-charge-neutralization conditions (an ALT ratio 0.05). Separate breakup is followed by erosive breakup, and the flocs become denser by incorporating the small fragments generated. Erosion breakup predominates and the floc granulates spherically. The granulation of flocs is related to the gelation of aluminum hydroxide gel.

In summary, we were able to clearly detect changes in the floc structure by conducting the test under a constant laminar flow. We are one step closer to elucidating the flocculation conditions under which flocs become denser and more compact. In the future, we plan to conduct tests at lower *G* values to investigate the phenomenon in more detail.

## 4. Materials and Methods

### 4.1. Couette Device and Photographic Equipment

Flocs had complex structures and were brittle and easily broken. To quantify the floc in the water, an imaging method was chosen, but some ingenuity was required to capture the sides of the rotating cylinder. To visualize the floc in the water, a preliminary test was first conducted with ceiling lighting, and a luminance distribution was observed in the vertical direction of a cylinder filled with test water. Since this vertical luminance distribution was fallacious because of erroneous capture of the particle size and shape of the floc, we decided to use background illumination [38]. There were some problems with imaging caused by the background illumination. For example, extremely small particles or abundant particles could not be detected, errors occurred in particle size determination due to their distribution in the depth direction, and the 3D shape, including the depth direction, could not be grasped. In addition, if there were multiple flocs within the depth of focus of the imaging sensor, they tended to appear as one floc when they were close together. Conversely, if the particles were sparsely clustered, even if they belonged to a single floc, they were erroneously counted as multiple particles as a result of the brightness threshold setting for analyses [39,40]. Despite these problems, particle measurement techniques using imaging with background illumination were well suited to the measurement of rapidly growing flocs. This is because flocs flow in water, grow from a few micrometers to several millimeters, and change their structure.

**Figure 7:**
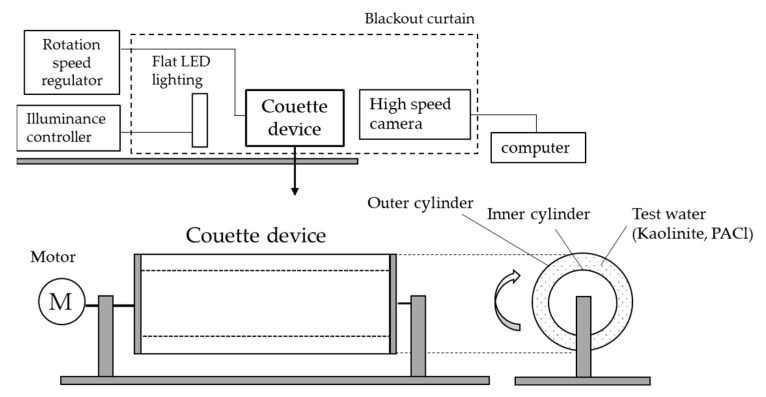
The test equipment consisted of the Couette device with horizontally rotating outer cylinder and a high-speed camera. Background lighting was used to capture clear images of floc size and shapes. The gap between the inner and outer cylinders generated a laminar shear flow in relation to the rotational speed of the outer cylinder.

Rotation tests to capture changes over time in the floc structure were performed using the Couette device, a double-cylinder device with a rotating outer cylinder placed horizontally and a high-speed camera (FASTCAM Mini AX50; Photron Ltd., Tokyo, Japan). Figure 7 shows the configuration of the test equipment. The Couette device was placed horizontally and a high-speed camera was fixed in front of it; LED flat lighting was placed behind it across the double cylinder. The Couette device was placed horizontally to avoid the effects of gravity settling. The Couette device and camera were covered with a blackout curtain to avoid the influence of ambient light. The gap between the inner and outer cylinders of the Couette device was filled with test water. The inner diameter of the outer cylinder was 11.5 cm, the outer diameter of the inner cylinder was 9 cm, and the gap was 1.25 cm. The length of the cylinder was 26 cm, and the volume of the gap was approximately 1 L. To stabilize the flow generated in the gap, the inner cylinder was fixed and only the outer cylinder was able to rotate. The Couette device had a motor for the rotation of the outer cylinder, and the speed could be adjusted from 1 rpm to 267 rpm by a speed controller (BXM series; Oriental Motor Co., Ltd., Tokyo, Japan). During the rotation, a constant upward shear force *τ* N/m^2^ was generated in the gap, as viewed from the camera side, by Newton’s viscosity law, *τ*:

(2) $$\tau =\mu \frac{U}{h}.$$

where *μ* represents the viscosity of water, *U* represents the velocity of movement of the wall surface, and *h* represents the distance from the wall surface. When the outer cylinder was rotated at 120 rpm and 160 rpm in this test, a constant shearing force of 0.058 N/m^2^ and 0.077 N/m^2^, respectively, was generated in the gap. Both the inner and outer cylinders were made of transparent acrylic, and the floc aggregation in the gap was photographed from the outside of the device by a high-speed camera. The target was a particle near the vertical center of the double cylinder, in the middle of the gap between the inner and outer cylinders. To minimize distortion effects, the camera was placed to have the light source, the center of the double cylinders, and the image sensor connected by a straight line, as well as the plane passing through the light source and axis of rotation parallel to the image sensor. Photographical targets were flocs in the gap on the camera side. A plastic film was attached to the inside of the inner cylinder so that flocs on the far side of the inner cylinder would not be reflected. The rotational speed of the outer cylinder *N* was adjustable, and we tested two conditions, namely 120 rpm and 160 rpm. The agitation intensity *G* in the gap was calculated as follows:

(3) $$G=\frac{2\pi NR}{60d_{gap}}.$$

where *R* represents the inner radius of the outer cylinder and *d_gap_* represents the gap between the inner and outer cylinders [41]. Photographical conditions for the rotation test were automatic, with a shutter speed of 1/10,000 s, a frame rate of 2000 fps., and a shooting frequency of once every 2 s. The start of rotation was set to 0 s, and the maximum recording time was 1200 s. The image size was 1024 pixels squares with a scale of 0.04122 mm/pixel (Figure 1b) and 0.04916 mm/pixel (all except Figure 1b). These scales were values determined by the distance between the high-speed camera and the cylinder and was measured using the following method. A stainless steel scale with a rectangular object attached to the back surface was fixed to the outer wall of the inner cylinder and filled with water. The number of pixels corresponding to 1 mm was calculated from an image taken of the scale in water.

### 4.2. Image Analysis and Data Statistics

Image analysis was performed using open-source public domain image processing software [42]. Images were captured in RGB format and converted to 8-bit format for analysis according to the specifications of the analysis software. The ROI was extracted for each test condition, with the regions having nearly same luminance tonal frequency. The main image analysis items were projected area *A*, perimeter length *L*, number of particles, and circularity *γ_c_*. Floc diameter *d* was a circle equivalent diameter (CED). The diameter *d* was converted from the projected area *A* as follows:

(4) $$d=2\sqrt{\frac{A}{\pi }}.$$

The circularity *γ_c_* was a shape indicator, *γ_c_*:

(5) $$\gamma_{c}=\frac{4\pi A}{L^{2}}.$$

and the closer it was to 1, the closer it was to being circular. The results of the analysis were assessed statistically, and the representative value for the floc diameter was *D*_90_, which is the 90% diameter of the cumulative frequency distribution on a volumetric basis. The structural change of the floc was represented by the relationship between *D*_90_ and the *GT* value. The *GT* value was the product of the agitation intensity *G* and the cumulative rotation time *T*, a dimensionless quantity that normalizes the agitation intensity and time received by the floc. Floc growth and breakup were indicated by the increase or decrease of *D*_90_ with the *GT* value. In a previous report [36], it was reported that *D*_90_ could be used to determine the typical trend of floc particle size change. Because of the background lighting, the flocs in the image appeared as a shadow. Although flocculation began immediately after the start of the test, the flocculation flocs in the very initial stage were so small that they were not clearly detectable by the image sensor.

### 4.3. Test Water Materials

**Figure 8:**
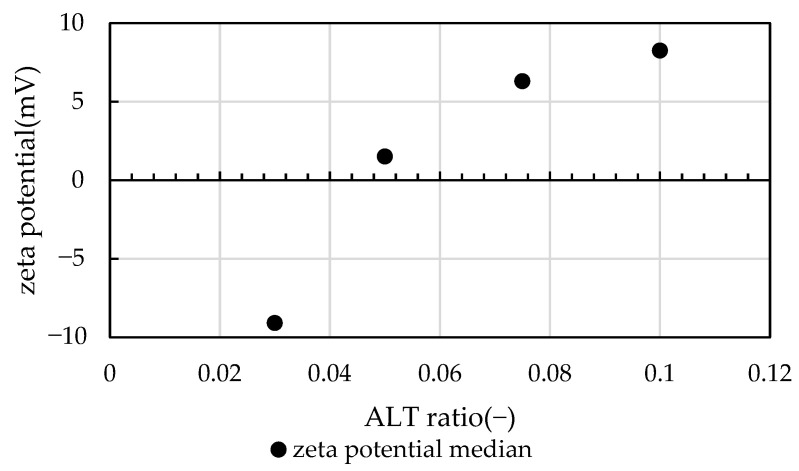
Zeta potential results of jar test supernatant water measured by the authors. The zeta potentials are reported as median values; an ALT ratio of 0.05 is close to charge neutralization.

Test water was simulated river water, and 100 mg/L kaolinite was dispersed in tap water from the University of Tsukuba. Kaolinite was a Si-based fine mineral particle with an average particle size of about 5 µm (measured by Laser diffraction particle size analyzer; SALD-3100, Shimadzu Co., Kyoto, Japan). Tap water had a pH of 7.4–7.5 and a water temperature of 20.0–23.0 °C. Tap, water was degassed in advance by ultrasonic suction deaeration for 10 min to prevent air bubbles from being generated during the rotation test which would interfere with imaging. The room temperature was always kept at 20.0 °C. The water temperature was 20.0 °C at the start of the test; due to the heat from an LED light source and the rotational motor, the water temperature gradually increased, reaching a maximum of 23.0 °C. Although water temperature affects flocculation, we considered that relative comparisons could be made for each condition. The flocculant was poly-aluminum chloride (PACl, 10.0% as Al_2_O_3_), which is commonly used in drinking water treatment in Japan. The advantages of PACl are better flocculation than aluminum sulfate and relatively easy floc formation even at low water temperatures [43]. Disadvantages are lighter flocs weight than iron-based flocculants and a narrow flocculation pH range of 6 to 8. PACl was pre-diluted with ultrapure water and added through the inlet of the outer casing of the Couette device filled with kaolinite suspension to achieve an injection rate *C_PACl_* of 30 to 100 mg/L. The ALT ratio was expressed as:

(6) $$ALT\;ratio=\frac{C_{PACl}R_{Al}}{C_{K}}.$$

using the concentration of kaolinite suspension *C_K_* and the aluminum content in the PACl *R_Al_*. *C_K_* was fixed and the ALT ratio was adjusted by changing *C_PACl_* with the ALT ratio of 0.03 to 0.10 as the test range. Figure 8 shows the zeta potential measurements (median) of the test water injected with PACl. A preliminary beaker-scale test to determine the flocculant injection rate, called the jar test, was performed to measure the zeta potential of the supernatant water after floc sedimentation. After the flocs were allowed to settle and separate for 1 h, the supernatant water was collected and measured using a zeta potential analyzer (Zeecom; Microtec Co., Ltd., Chiba, Japan). ALT ratios of 0.03–0.10 were within the zeta potential range of ±10 mV, where flocculation easily occurs. The ALT ratio of 0.03 had a negative zeta potential and was slightly deficient in flocculant, the ALT ratio of 0.05 had a zeta potential near 0, which was an appropriate condition for near charge neutralization, and the ALT ratio of 0.10 had a positive zeta potential and was considered overdose. After the experiment, the pH was 7.44 at an ALT ratio of 0.03, 7.23 at an ALT ratio of 0.05, and 7.04 at an ALT ratio of 0.10.

## Data Availability

The data that support the findings of the current study are listed within the article.
